# Wet Chemical Synthesis of Non-solvated Rod-Like α'-AlH_3_ as a Hydrogen Storage Material

**DOI:** 10.3389/fchem.2019.00892

**Published:** 2020-01-15

**Authors:** Haizhen Liu, Hongyu Ma, Longfei Zhang, Shichao Gao, Xinhua Wang, Li Xu, Shuangyu Liu, Xiantun Huang, Chenglin Lu, Hui Luo, Hua Ning, Zhiqiang Lan, Jin Guo

**Affiliations:** ^1^Guangxi Colleges and Universities Key Laboratory of Novel Energy Materials and Related Technology, Guangxi Novel Battery Materials Research Center of Engineering Technology, School of Physical Science and Technology, Guangxi University, Nanning, China; ^2^State Key Laboratory of Silicon Materials, School of Materials Science and Engineering, Zhejiang University, Hangzhou, China; ^3^State Key Laboratory of Advanced Power Transmission Technology, Global Energy Interconnection Research Institute Co. Ltd., Beijing, China; ^4^Department of Materials Science and Engineering, Baise College, Baise, China

**Keywords:** hydrogen storage, aluminum hydride, synthesis, desolvation, desorption

## Abstract

Aluminum hydride (AlH_3_) is a promising candidate for hydrogen storage due to its high hydrogen density of 10 wt%. Several polymorphs of AlH_3_ (e.g., α, β, and γ) have been successfully synthesized by wet chemical reaction of LiAlH_4_ and AlCl_3_ in ether solution followed by desolvation. However, the synthesis process of α'-AlH_3_ from wet chemicals still remains unclear. In the present work, α'-AlH_3_ was synthesized first by the formation of the etherate AlH_3_ through a reaction of LiAlH_4_ and AlCl_3_ in ether solution. Then, the etherate AlH_3_ was heated at 60°C under an ether gas atmosphere and in the presence of excess LiAlH_4_ to remove the ether ligand. Finally, α'-AlH_3_ was obtained by ether washing to remove the excess LiAlH_4_. It is suggested that the desolvation of the etherate AlH_3_ under an ether gas atmosphere is essential for the formation of α'-AlH_3_ from the etherate AlH_3_. The as-synthesized α'-AlH_3_ takes the form of rod-like particles and can release 7.7 wt% hydrogen in the temperature range 120–200°C.

## Introduction

Aluminum hydride (AlH_3_) is a kinetically stable metal hydride under ambient conditions. It theoretically has a high hydrogen capacity of 10 wt% and can release hydrogen at temperatures below 200°C (Sandrock et al., 2005; Graetz, 2009; Graetz et al., 2011). Therefore, it has long been considered as a promising hydrogen storage media for on-board applications. There are seven known polymorphs of AlH_3_: α-, α'-, β-, γ-, δ-, ε-, and ζ-AlH_3_ (Brower et al., 1976). These AlH_3_ polymorphs have different structures and thermal stabilities and thus have slightly different decomposition properties and mechanisms. α-AlH_3_ is the most stable polymorph and will undergo direct decomposition to form Al and H_2_ with an increase in temperature (Sandrock et al., 2005; Graetz and Reilly, 2006; Orimo et al., 2006). The other polymorphs, such as β-AlH_3_ and γ-AlH_3_, will first transform into the more stable α-AlH_3_ and then decompose to form Al and H_2_ (Graetz and Reilly, 2006). Direct decompositions of γ-AlH_3_ and α'-AlH_3_ to form Al and H_2_ without the first phase transition have also been reported in the literature (Sartori et al., 2008; Liu et al., 2013; Gao et al., 2017).

The synthesis of AlH_3_ dates back to 1942 when Stecher and Wiberg (1942) prepared the AlH_3_ amine complex in an impure form. The synthesis method of AlH_3_ was then modified and improved by other researchers (Finholt et al., 1947; Chizinsky et al., 1955; Ashby, 1964). In 1976, Brower et al. (1976) summarized their findings on the synthesis of non-solvated AlH_3_ by the wet chemical method. They used LiAlH_4_ and AlCl_3_ as the starting materials and ether as the solvent. Generally, LiAlH_4_ was reacted with AlCl_3_ in the ether solution to form AlH_3_·*n*Et_2_O and LiCl [reaction (1)]. The precipitate LiCl was then removed by filtration, and the AlH_3_·*n*Et_2_O precipitated slowly during storage. The obtained solid, AlH_3_·*n*Et_2_O, was heated under certain conditions to remove the ether ligand [reaction (2)], which was called the desolvation process. Depending on the desolvation conditions used, AlH_3_ would crystalize in different structures.

(1) $$\begin{array}{l} 3\text{LiAl}{\text{H}}_{4}+\text{AlC}{\text{l}}_{3}\overset{ether}{\to }4\text{Al}{\text{H}}_{3}\cdot \text{nE}{\text{t}}_{2}\text{O}+3\text{LiCl↓} \end{array}$$

(2) $$\begin{array}{l} \text{Al}{\text{H}}_{3}\cdot \text{nE}{\text{t}}_{2}\text{O}\to \text{Al}{\text{H}}_{3}+\text{ether}↑ \end{array}$$

Non-solvated α-, β-, and γ-AlH_3_ have been successfully synthesized by the wet chemical method (Brinks et al., 2006, 2007a,b; Graetz and Reilly, 2006; Orimo et al., 2006; Liu et al., 2013; Gao et al., 2017). These are the polymorphs of AlH_3_ that have been intensively studied. However, the intrinsic decomposition properties of α'-AlH_3_ are still unclear due to the fact that pure and non-solvated α'-AlH_3_ is hard to synthesyze. As far as we know, the synthesis of pure and non-solvated α'-AlH_3_ by the wet chemical method has not yet been reported in the open literature. Although Brower et al. (1976) suggested that α'-AlH_3_ can be synthesized by the slow desolvation of AlH_3_·*n*Et_2_O, no characterized product of α'-AlH_3_ was disclosed.

In 2006, Brinks et al. (2006) utilized the cryomilling method to prepare α'-AlD_3_ from a mixture of 3LiAlD_4_ + AlCl_3_. It was shown that cryomilling at a temperature as low as 77 K resulted in the formation of only AlD_3_ and LiCl. The AlD_3_ obtained was a mixture of 2/3α-AlD_3_ + 1/3α'-AlD_3_ (Brinks et al., 2006). Another work by Sartori et al. (2009) showed that the yield of AlD_3_ was increased by using 3NaAlH_4_ + AlCl_3_ or 3LiAlD_4_ +AlBr_3_ as the raw materials. In addition, the relative amount of α'-AlD_3_ over α-AlD_3_ was increased from 0.63–0.67 to 1.05 by the addition of FeF_3_ into the 3LiAlD_4_ + AlCl_3_ mixture. Although α'-AlH_3_ can be obtained by the cryomilling method, the unwanted product of LiCl salt is difficult to remove. Moreover, the α'-AlH_3_ prepared by this method is usually accompanied by α-AlH_3_ polymorphs.

In the present work, the synthesis of non-solvated and pure α'-AlH_3_ by the wet chemical method is studied. The decomposition properties of α'-AlH_3_ will also be preliminarily revealed.

## Experimental Details

### Synthesis of α'-AlH_3_

The synthesis process of α'-AlH_3_ employed here is similar to that reported by Brower et al. (1976). However, some conditions needed to be modified. In detail, 1 M ether (Sinopharm Group, Analytical purity) solution of LiAlH_4_ (TCI, 98% purity) was mixed with 1 M ether solution of AlCl_3_ (Aldrich, 99.99% purity) at a molar ratio of 4:1. It should be noted that LiAlH_4_ was used in excess. Brower et al. (1976) found that the etherate AlH_3_ will decompose to Al if heated under a vacuum, but, in the presence of excess LiAlH_4_, the ether can be removed without decomposition. LiAlH_4_ will react with AlCl_3_ upon mixing in the ether solution to form the etherate AlH_3_ (AlH_3_·*n*Et_2_O) and LiCl precipitate based on reaction (3). The mixed solution was stirred for 2 min to ensure that the reaction was completed. Immediately after that, the LiCl precipitate was removed by filtration and the liquid ether was removed by slowly evacuation at room temperature. The dry and white residue obtained, which was a mixture of 4AlH_3_·*n*Et_2_O + LiAlH_4_, was ground to powder with a mortar and pestle for heating treatment. Powder samples were then heated at certain temperatures for various durations under certain atmospheres to remove the ether ligand [reaction (4)]. The conditions used for heat treatment significantly impact the desolvation products of the 4AlH_3_·*n*Et_2_O + LiAlH_4_ mixture, as will be shown in the next section. Finally, the desolvated 4AlH_3_·*n*Et_2_O + LiAlH_4_ mixture was ether-washed to remove the excess LiAlH_4_, and AlH_3_ was obtained.

(3) $$\begin{array}{l} 4\text{LiAl}{\text{H}}_{4}+\text{AlC}{\text{l}}_{3}\overset{ether}{\to }4\text{Al}{\text{H}}_{3}\cdot \text{nE}{\text{t}}_{2}\text{O}+\text{LiAl}{\text{H}}_{4}+3\text{LiCl}↓ \end{array}$$

(4) $$\begin{array}{l} 4\text{Al}{\text{H}}_{3}\cdot \text{nE}{\text{t}}_{2}\text{O}+\text{LiAl}{\text{H}}_{4}\to \text{Al}{\text{H}}_{3}+\text{LiAl}{\text{H}}_{4}+\text{ether}↑ \end{array}$$

### Characterizations of α'-AlH_3_

Powder X-ray diffraction (XRD, PANalytical X'Pert Pro, Cu Kα, 40 kV, 40 mA) was used to study the phase structures of the samples. The samples for XRD studies were sealed with an amorphous membrane to protect them from oxidation during the sample transformations and measurements. Scanning electronic microscopy (SEM, FEI SIRION-100, 25 kV) was used to study the morphology of the as-synthesized α'-AlH_3_. The hydrogen desorption property of the as-synthesized α'-AlH_3_ was studied by using a home-made Sieverts-type hydrogen sorption measurement apparatus based on the volumetric method. Experimentally, the samples were sealed in a reactor and were heated under an initial vacuum gradually from room temperature to the set temperature with a heating rate of 2°C/min.

## Results and Discussion

On the synthesis of AlH_3_ by the wet chemical reaction in the ether solution, the conditions (desolvation aid, temperature, time, atmosphere) used in the desolvation stage [reaction (4)] significantly affect the desolvation product of AlH_3_·*n*Et_2_O (Brower et al., 1976). α-AlH_3_ can be obtained by heating the AlH_3_·*n*Et_2_O at 60–80°C under a vacuum in the presence of excess LiAlH_4_ and LiBH_4_, while γ-AlH_3_ is formed when the AlH_3_·*n*Et_2_O is heated at 60–70°C under a vacuum in the presence of only excess LiAlH_4_ (Brower et al., 1976). It should be noted that the AlH_3_·*n*Et_2_O should be desolvated in the presence of excess LiAlH_4_ (and LiBH_4_), with which AlH_3_·*n*Et_2_O can easily transform to AlH_3_ without decomposition (Brower et al., 1976).

**Figure 1:**
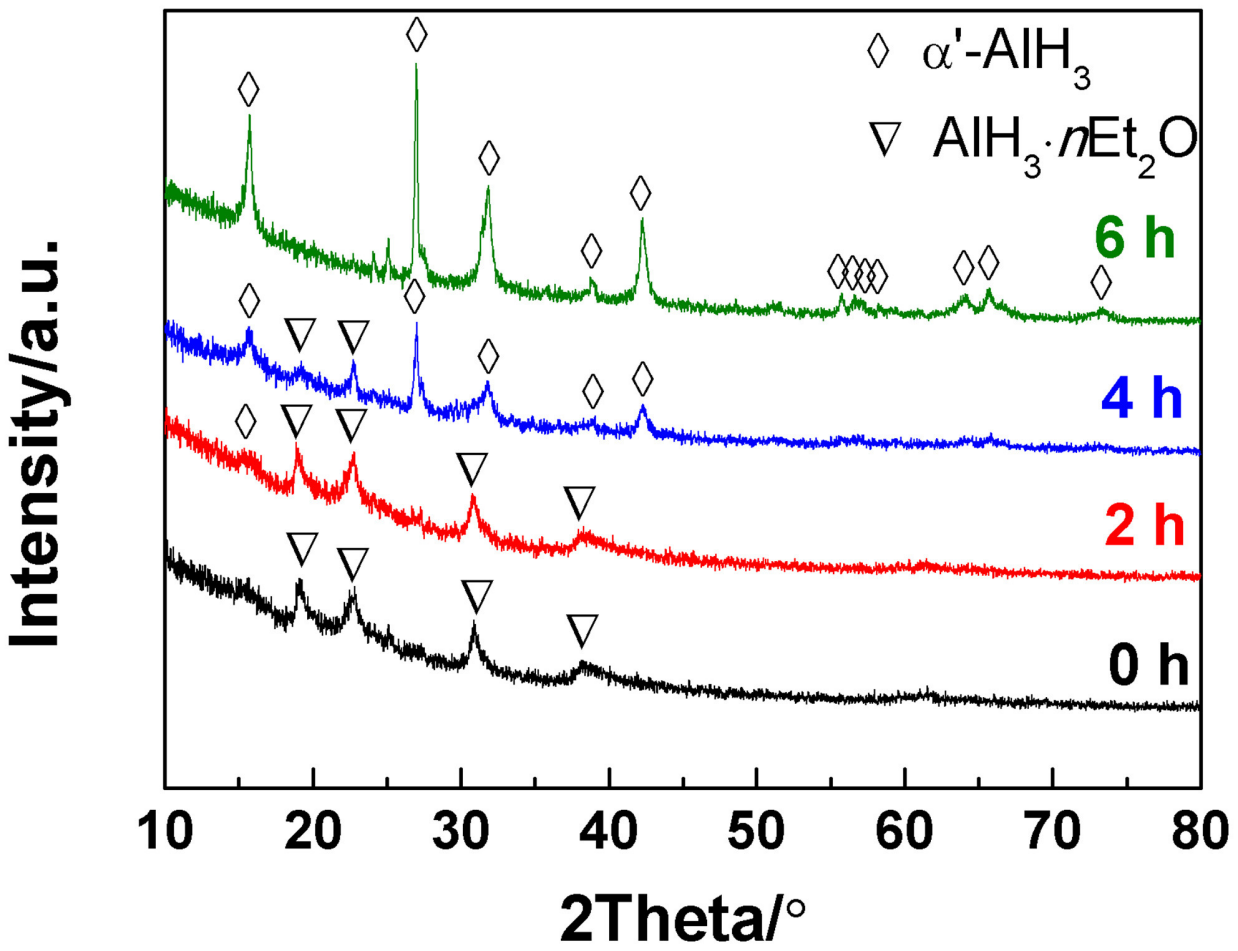
XRD patterns of the desolvation products of AlH_3_·*n*Et_2_O heated at 60°C for various durations.

In the present work, the AlH_3_·*n*Et_2_O was heated under a gaseous ether atmosphere, which is the key factor for producing α'-AlH_3_. The ether atmosphere was generated by injecting a drop of liquid ether into the sample reactor. The liquid ether can easily transform to gaseous ether during heating to 60–80°C since the boiling point of ether is as low as 34.6°C. In this way, the AlH_3_·*n*Et_2_O can undergo desolvation under a gaseous ether atmosphere. Figure 1 shows the XRD patterns of the desolvation products of AlH_3_·*n*Et_2_O heated at 60°C for various durations under an atmosphere of gaseous ether. It can be seen that traces of α'-AlH_3_ formed after desolvation for 2 h. With an increase in the desolvation duration, more and more α'-AlH_3_ formed. The AlH_3_·*n*Et_2_O can totally transformed to α'-AlH_3_ after desolvation for 6 h.

**Figure 2:**
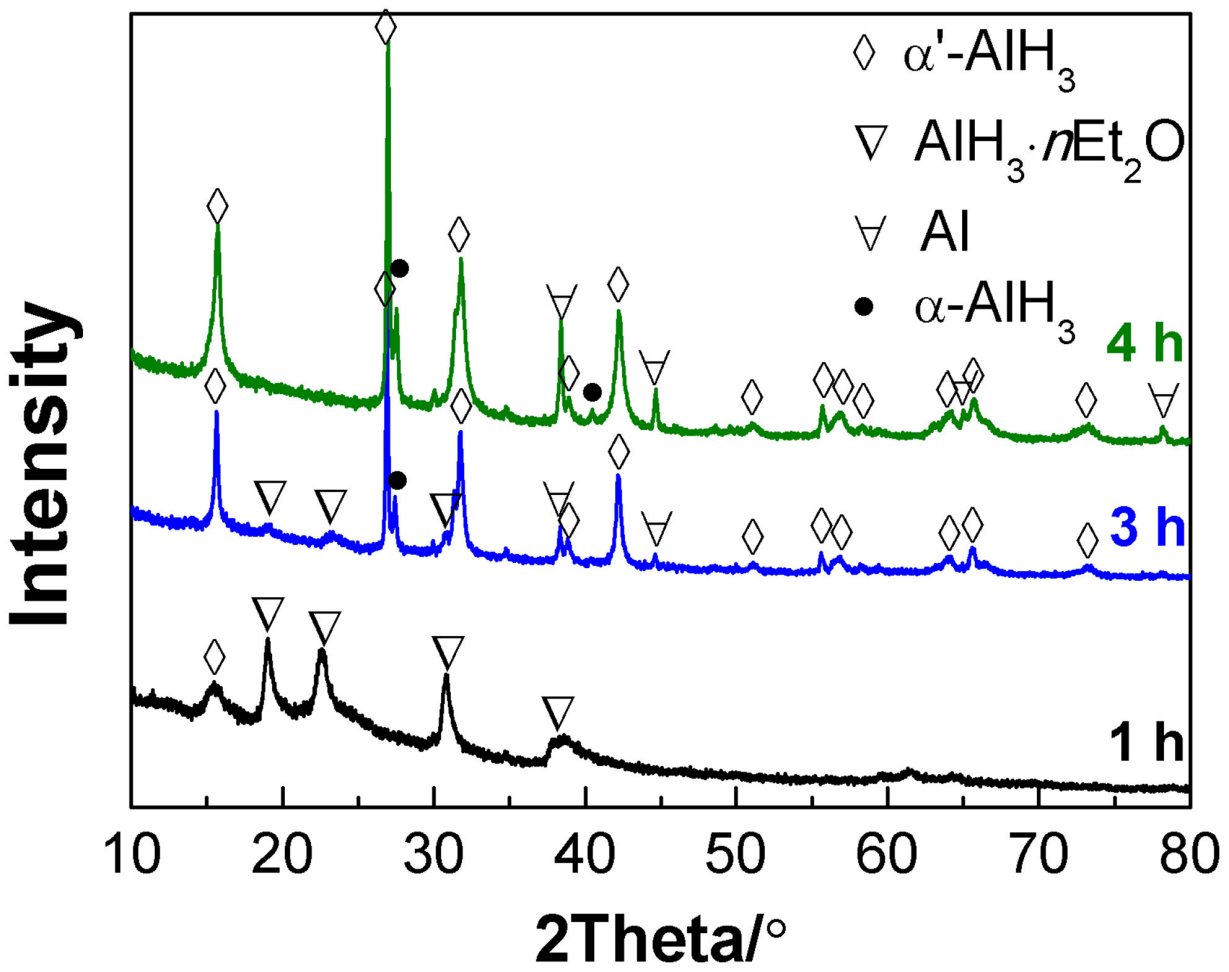
XRD patterns of the desolvation products of AlH_3_·*n*Et_2_O heated at 75°C for various durations.

When the desolvation of the AlH_3_·*n*Et_2_O was conducted at 75°C, the transformation to α'-AlH_3_ proceeded more rapidly. Figure 2 shows the XRD patterns of the desolvation products of AlH_3_·*n*Et_2_O after heating at 75°C for various durations under an atmosphere of gaseous ether. It was observed that some traces of α'-AlH_3_ formed after desolvation for only 1 h. After 4 h of desolvation, the AlH_3_·*n*Et_2_O had completely transformed to AlH_3_, which was a mixture of α'-AlH_3_ and α-AlH_3_. This means that some of the α'-AlH_3_ may have transformed into more stable α-AlH_3_ during heat treatment at 75°C. Therefore, a lower desolvation temperature (e.g., 60°C) is preferred in order to produce pure α'-AlH_3_.

**Figure 3:**
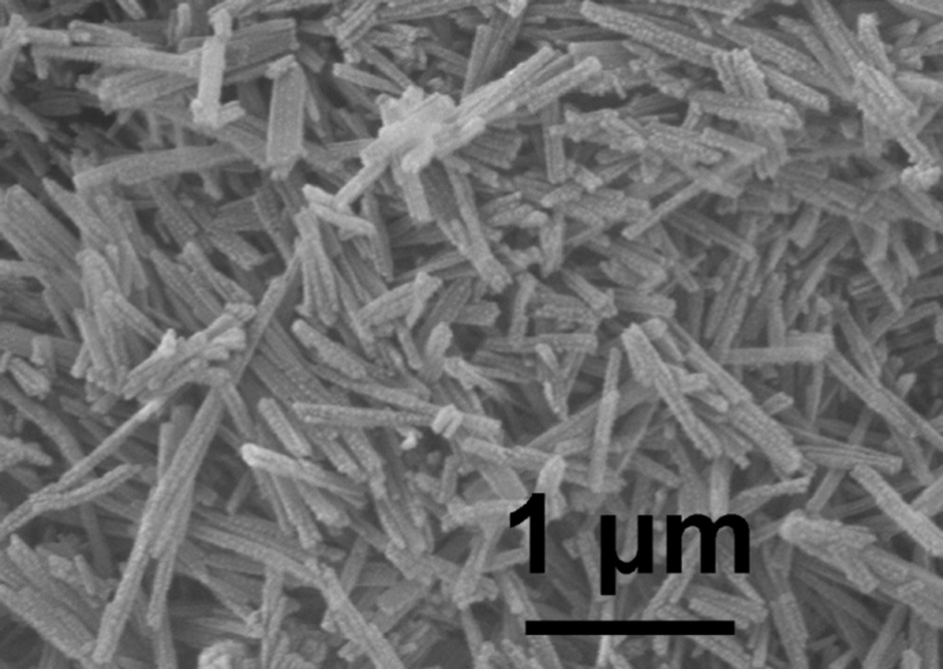
SEM image of the as-synthesized α'-AlH_3_.

The morphology of the as-synthesized α'-AlH_3_ was studied by SEM techniques, as shown in Figure 3. It can be seen that the as-synthesized α'-AlH_3_ takes the form of rod-like particles with lengths of about 1 μm and widths of about 100 nm. This unique particle morphology may benefit the hydrogen desorption process of α'-AlH_3_ because it possesses more surface area than other morphologies such as spheres of similar dimensions.

**Figure 4:**
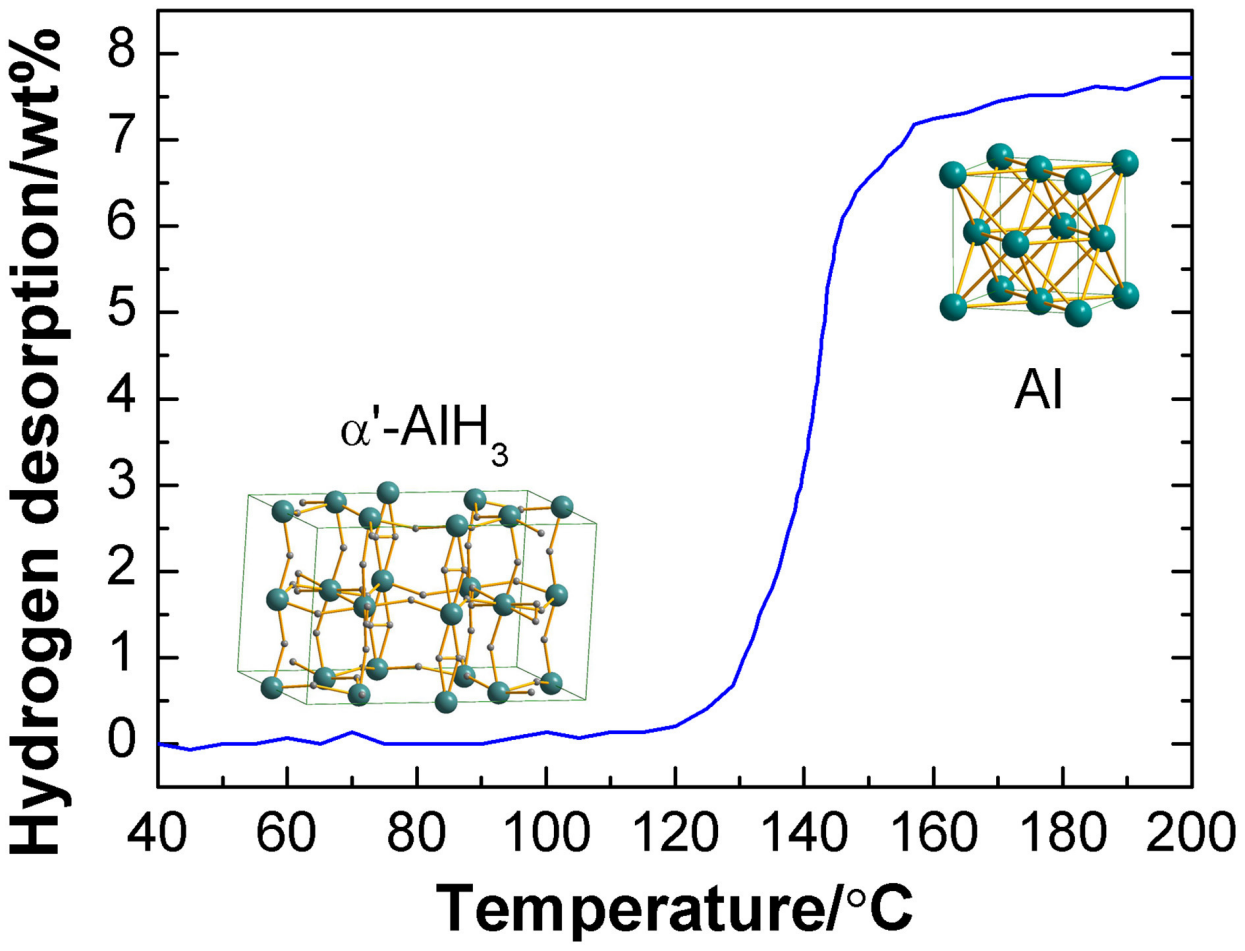
Hydrogen desorption curve of the as-synthesized α'-AlH_3_ at a heating rate of 2°C/min.

The hydrogen desorption curve of the as-synthesized α'-AlH_3_ with a heating rate of 2°C/min is shown in Figure 4. As can be seen that it starts to release hydrogen at 120°C and reaches a hydrogen desorption capacity of 7.7 wt% when the temperature is increased to 200°C. After hydrogen desorption, Al is formed. It should be noted that the practical capacity is somewhat lower than the theoretical value, which may be due to the impurity of the sample. This decomposition temperature range is similar to that of α-AlH_3_ and γ-AlH_3_ (Graetz and Reilly, 2006; Liu et al., 2013).

## Conclusion

Non-solvated α'-AlH_3_ was successfully synthesized by the wet chemical reaction of LiAlH_4_ and AlCl_3_ in ether solution followed by desolvation. The conditions used in the desolvation stage are the essential factors in producing α'-AlH_3_. Desolvation under a gaseous ether atmosphere is the key to the transformation of AlH_3_·*n*Et_2_O into non-solvated α'-AlH_3_. The as-synthesized α'-AlH_3_ particles are rod-like and can release 7.7 wt% hydrogen in the temperature range 120–200°C. The purity of the α'-AlH_3_ needs to be improved in future work.

## Data Availability Statement

All datasets generated for this study are included in the article/supplementary material.

## Author Contributions

All authors listed have made a substantial, direct and intellectual contribution to the work, and approved it for publication.

### Conflict of Interest

LX and SL were employed by the company Global Energy Interconnection Research Institute Co., Ltd. The remaining authors declare that the research was conducted in the absence of any commercial or financial relationships that could be construed as a potential conflict of interest.
